# A Case of APC Gene Mutation‐Associated Familial Adenomatous Polyposis With Multiple System Malignancies

**DOI:** 10.1002/cnr2.70362

**Published:** 2025-10-17

**Authors:** Ren Yijing, Wang Wenjun, Gao Xiang, Xing Kongling, Chen Yunxia, Liao Wei, Zhou Ping

**Affiliations:** ^1^ Second Ward, Department of Radiotherapy, the First Affiliated Hospital, the First School of Clinical Medicine Hainan Medical University Haikou China; ^2^ Key Laboratory of Emergency and Trauma of Ministry of Education, Engineering Research Center for Hainan Biological Sample Resources of Major Diseases & the Hainan Branch of National Clinical Research Center for Cancer & the First Affiliated Hospital Hainan Medical University Haikou China; ^3^ Research Transformation Department Hainan Free Trade Port Health Medicine Research Institute Baoting China; ^4^ Department of Respiratory Medicine, Hongsen Hospital Harbin Medical University Sanya China; ^5^ Second Ward, Department of Radiotherapy, Key Laboratory of Emergency and Trauma of Ministry of Education, the First Affiliated Hospital Hainan Medical University Haikou China

**Keywords:** APC gene, colorectal cancer, familial adenomatous polyposis, thyroid cancer

## Abstract

**Background:**

Familial adenomatous polyposis (FAP) is an autosomal dominant inherited disorder, with a nearly 100% risk of developing colorectal cancer by the age of 40. The primary gene mutated in FAP is APC, and mutations in certain regions of the APC gene may be associated with thyroid disorders, including malignant neoplasms, benign nodules, and endocrine diseases of the thyroid. FAP‐associated colorectal cancer (FAP‐CRC) demonstrates poorer treatment outcomes compared to sporadic colorectal cancer, which may be attributed to several factors such as distinct molecular pathogenesis, chromosomal instability (CIN), and tumor microenvironment (TME).

**Case:**

We describe the case of a 30‐year‐old female patient with a history of papillary thyroid carcinoma who presented with abdominal pain. Gastrointestinal endoscopy revealed multiple polyps in the stomach and colon. Additionally, the patient was found to have metastatic colorectal cancer with hepatic and pulmonary involvement. Further genetic testing revealed a deletion mutation in the APC gene at exon 16, c.1974_1975del (p.Asn659Glnfs*14). Despite the implementation of multiple therapeutic regimens, the patient's condition showed a poor response, ultimately leading to her demise. We conducted an in‐depth analysis of the potential factors contributing to this outcome.

**Conclusion:**

APC gene mutations lead to FAP and subsequent colorectal cancer, and may also predispose individuals to thyroid disorders, including malignancies, benign nodules, and endocrine dysfunction. Therefore, we recommend that young patients diagnosed with thyroid cancer undergo a thorough evaluation of family history for hereditary conditions. Additionally, consideration should be given to gastrointestinal endoscopic and ophthalmologic screening, as well as molecular genetic testing. When multiple gastric and colorectal polyps are detected, genetic alterations in APC or MUTYH should be suspected. In particular, female patients diagnosed with FAP before the age of 31 should undergo annual thyroid ultrasound surveillance.

## 1 Introduction

Familial adenomatous polyposis (FAP), an autosomal dominant inherited colorectal disorder, is caused by mutations in the APC gene. Its clinical hallmark is the development of multiple colorectal adenomatous polyps, with the number ranging from tens to thousands. Based on polyp burden, FAP is classified into attenuated FAP (AFAP), characterized by 10–100 polyps; classical FAP (CFAP), defined by 100–1000 polyps; and severe FAP (SFAP), presenting with more than 1000 polyps. Regarding age at onset/diagnosis, patients with AFAP are typically > 40 years old at disease manifestation or diagnosis, whereas those with CFAP or SFAP are usually < 40 years old [1]. In classical FAP (CFAP), the first adenomatous polyp typically develops during the second decade of life, indicating that the initial polyp arises as early as age 15 in approximately half of affected individuals. By age 35, nearly 95% of patients with FAP exhibit polyps. In the absence of timely diagnosis and intervention, the risk of developing colorectal cancer approaches 100% by age 40 [2].

It is important to avoid confusion with another form of adenomatous polyposis known as MUTYH‐associated polyposis (MAP), which is an autosomal recessive disorder caused by biallelic pathogenic variants in the MUTYH gene. Although both FAP and MAP present clinically with multiple colorectal adenomas and may exhibit overlapping polyp counts—particularly resembling attenuated FAP (AFAP)—they are fundamentally distinct entities. MAP is an autosomal recessive disorder characterized by the absence of APC gene mutations, but the presence of biallelic pathogenic variants in the MUTYH gene. MUTYH is a base excision repair gene located on chromosome 1p34.3‐p32.1. Mutations in this gene lead to G‐to‐T transversions in multiple genes, including APC and KRAS, within somatic cells. Compared with the general population, individuals with MAP have a 43%–63% risk of developing colorectal cancer by age 60, and the lifetime risk in unmonitored patients ranges from 80% to 90% [3]. MAP‐associated polyps are predominantly located in the right colon. In addition to colonic adenomas, the clinical phenotype commonly includes sessile serrated lesions and hyperplastic polyps. Furthermore, the clinical presentation of MAP is broader and more variable in its manifestations. Overall, MAP is characterized by a lower polyp burden, later onset of colorectal cancer, and a milder disease course compared to classical FAP, resembling attenuated FAP (AFAP) in phenotype. However, individuals with MAP may have an increased predisposition to extracolonic malignancies resembling those seen in Lynch syndrome, including ovarian, endometrial, urothelial, and skin cancers, as well as thyroid cancer, breast cancer, and sebaceous adenomas [4]. In some cases, MAP may present more severely, with patients developing a greater number of polyps. Due to the high mutational burden and frequent involvement of the KRAS gene, polyps in MAP may exhibit accelerated oncogenesis [5].

Outside of the GI tract, manifestations of FAP primarily include osteomas, dental abnormalities, congenital hypertrophy of the retinal pigment epithelium (CHRPE), epidermoid cysts, lipomas, fibromas, fibromatosis, medulloblastoma, and hepatoblastoma [2]. Furthermore, patients with FAP have an increased risk of developing other malignancies and benign tumors, including thyroid cancer, bilateral renal cancer, hepatoblastoma, pancreatic cancer, brain tumors, and adrenal adenomas. Notably, approximately 90% of FAP patients develop congenital hypertrophy of the retinal pigment epithelium (CHRPE), while about 10%–20% of patients are affected by desmoid tumors (also known as aggressive fibromatosis) [6]. Notably, congenital hypertrophy of the retinal pigment epithelium (CHRPE) is a benign retinal lesion that can be identified via fundoscopic examination. These lesions are characterized by well‐demarcated, darkly pigmented, flat or slightly elevated, round or oval areas, which may occur as solitary or multifocal lesions [7]. CHRPE is essentially characterized by localized hyperplasia and pigment accumulation of retinal pigment epithelial cells. Multiple and bilateral lesions exhibit the highest specificity for FAP. Therefore, some scholars propose that CHRPE may serve as a rapid, non‐invasive, and highly specific biomarker for FAP [8]. We identified a unique case of FAP through molecular genetic testing, in which the patient harbored a deletion mutation in the APC gene at exon 16, c.1974_1975del (p.Asn659Glnfs*14), a variant not previously reported in the literature. Additionally, the patient had neither congenital hypertrophy of the retinal pigment epithelium (CHRPE) nor desmoid tumors and developed thyroid cancer prior to the diagnosis of polyposis.

## 2 Case Description

**FIGURE 1:**
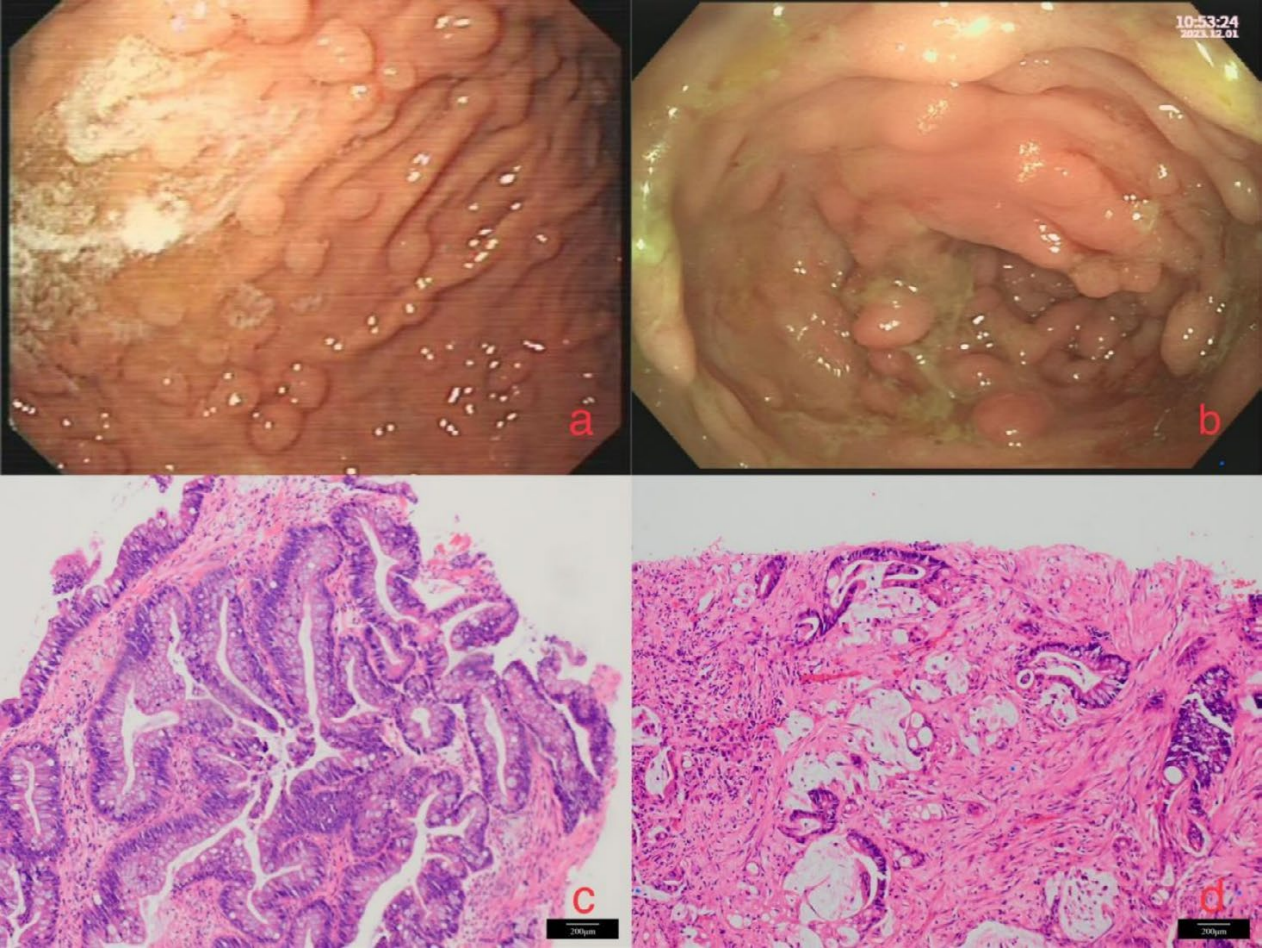
(a) Gastroscopy reveals multiple gastric polyps widely distributed across the gastric wall. (b) Colonoscopy shows variably sized colonic polyps with clustered distribution. (c) High‐power view (40×, H&E staining, Scale bar: 200 μm) of the colonic mass demonstrates dysplastic elongated nuclei, focal stratification, irregular glandular architecture, and stromal lymphoplasmacytic infiltration, consistent with adenocarcinoma. (d) High‐power view (40×, H&E staining, Scale bar: 200 μm) of the liver biopsy reveals infiltrative dysplastic glands and scattered tumor cells within normal hepatic parenchyma, exhibiting enlarged hyperchromatic nuclei.

A 30‐year‐old female presented to the outpatient gastroenterology clinic at the First Affiliated Hospital of Hainan Medical University in October 2023 with a one‐month history of recurrent abdominal pain. Accompanying symptoms included nausea and vomiting, which were non‐projectile in nature and occurred five times. The vomitus consisted of gastric contents. The patient also reported having scanty, loose stools approximately three times per day. She had self‐administered medications including “Fengliao Gastrointestinal Health, Smecta (Diosmectite Powder), and Saccharomyces boulardii lyo powder,” but her symptoms did not improve significantly, leading to her presentation at our clinic. Relevant medical history revealed that the patient underwent radical surgery for papillary thyroid carcinoma (PTC) at the age of 18. In January 2023, she underwent gastroscopy, which revealed “gastric polyposis” (Figure 1a). A detailed family history indicated that both her mother and maternal uncle had died from colorectal cancer.

**FIGURE 2:**
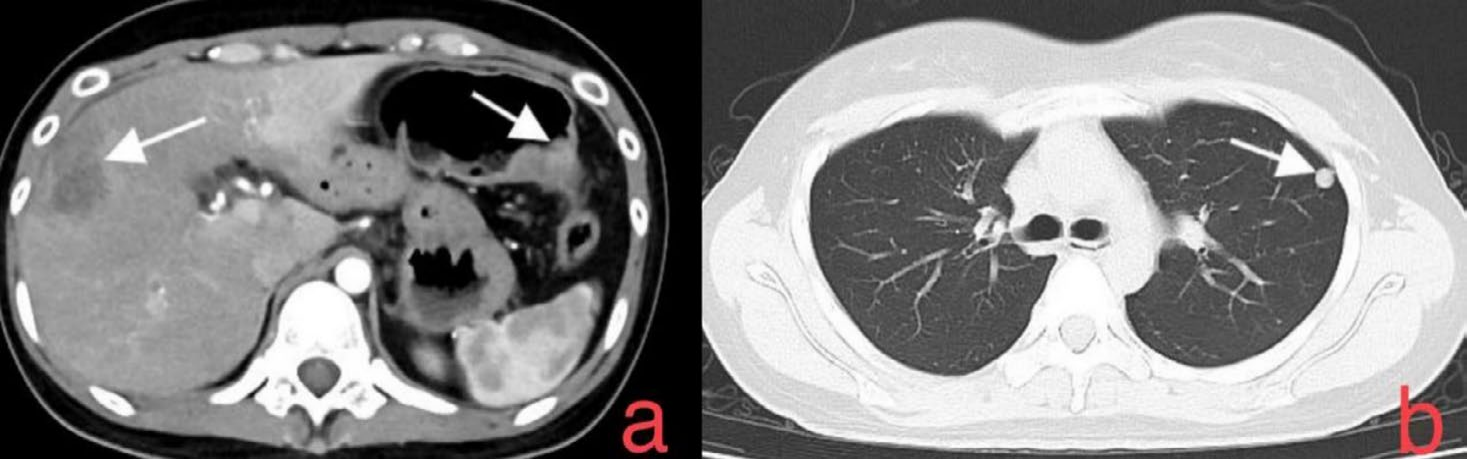
(a) Contrast‐enhanced abdominal CT scan showing a mass in the anterior segment of the right hepatic lobe (left arrow), exhibiting heterogeneous ring‐like enhancement and irregular wall thickening with focal luminal narrowing at the splenic flexure of the colon (right arrow), consistent with colorectal cancer and secondary incomplete colonic obstruction. (b) Chest CT scan showing a round mass in the apicoposterior segment of the left upper lobe (arrow), suggestive of metastatic carcinoma.

We performed contrast‐enhanced computed tomography (CT) of the chest and whole abdomen, which revealed the following findings:

- Irregular wall thickening and focal luminal narrowing at the splenic flexure of the colon, with a maximum thickness of approximately 13 mm, consistent with colorectal cancer causing incomplete obstruction of the proximal colon and small intestine (Figure 2a);
- A space‐occupying lesion in the anterior segment of the right hepatic lobe, with lobulated margins and measuring approximately 32 × 57 mm at its largest dimension. The lesion showed heterogeneous, ring‐like enhancement on contrast administration, highly suggestive of a metastatic tumor (Figure 2a);
- An additional small nodule in the caudate lobe of the liver, approximately 5 mm in diameter, likely represents another metastatic deposit;
- Nodules in the left upper lobe (apicoposterior segment) and the right lower lobe (posterior basal segment), which, in conjunction with the clinical history, are considered metastatic lesions (Figure 2b);
- Enlarged retroperitoneal lymph nodes with a short‐axis diameter of approximately 13 mm, consistent with metastatic involvement.

Due to the presence of obstruction proximal to the splenic flexure of the colon, we performed endoscopic placement of a colonic stent to relieve the obstruction. During the procedure, we obtained biopsy specimens from suspicious areas for histopathological evaluation. Colonoscopy revealed multiple polyps throughout the colorectum (Figure 1b). Histopathological examination of the colonic tissue revealed high‐grade intraepithelial neoplasia with superficial architectural involvement, which, in conjunction with the endoscopic findings, is consistent with adenocarcinoma. Immunohistochemical results: intact expression for MLH1, PMS2, MSH2, and MSH6, Ep‐CAM(+), Ki‐67 (hotspot area 90% +) (Figure 1c). For the hepatic mass, we performed a percutaneous liver biopsy. Histopathological examination confirmed adenocarcinoma. Immunohistochemical analysis revealed the following profile: HepPar‐1 (−), CK7 (−), CK19 (+), CK20 (+), Villin (+), CK18 (+), CDX2 (+), Ki‐67 (active area 70% +), and SATB2 (+). Integrating the clinical, morphological, and immunohistochemical findings, the tumor is consistent with metastatic adenocarcinoma of colorectal origin (Figure 1d). Given the patient's history of thyroid cancer, we performed a thyroid ultrasound which indicated “post‐thyroidectomy, with no significant abnormalities noted in the bilateral cervical lymph nodes.”

**FIGURE 3:**
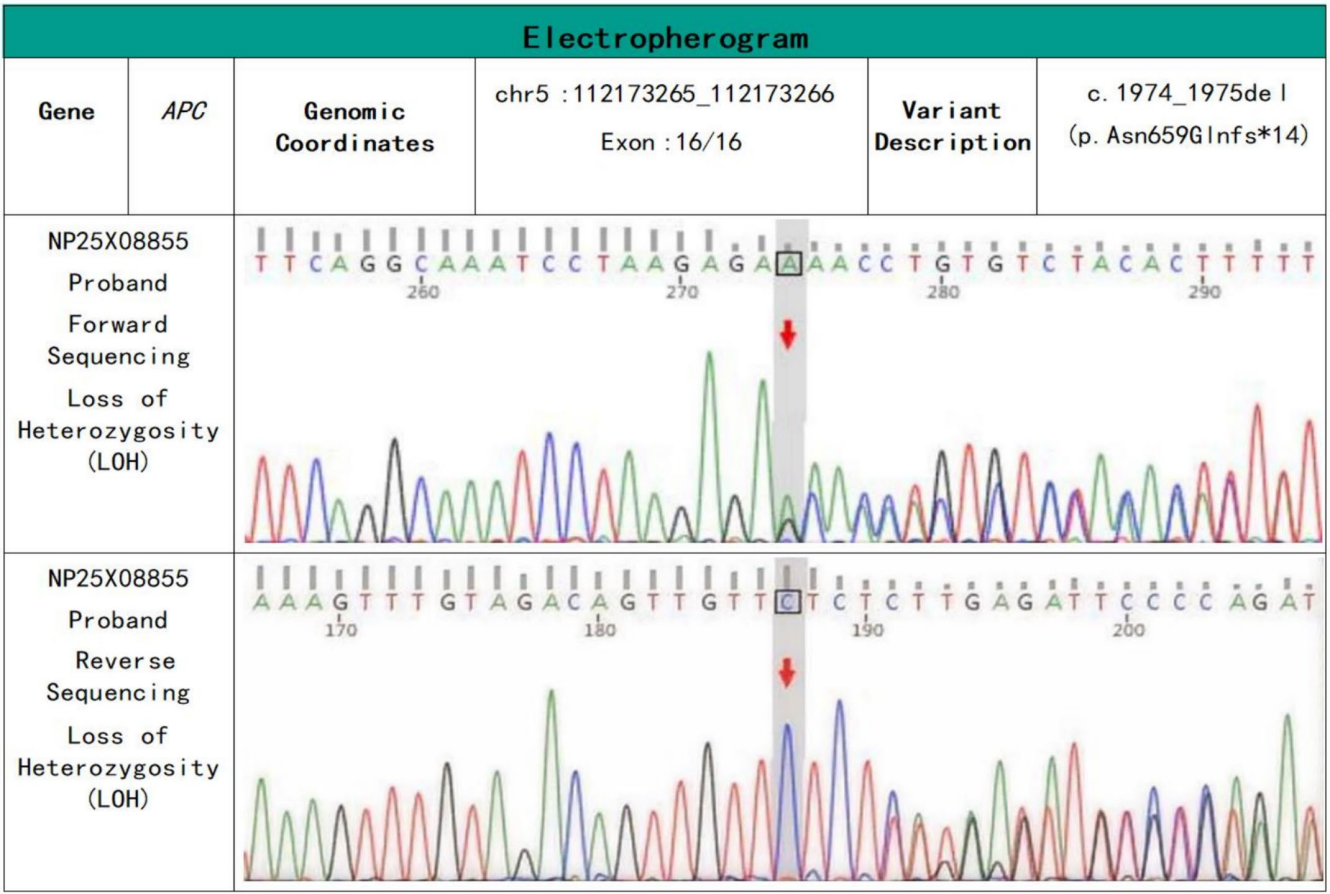
Sanger sequencing chromatogram showing a heterozygous deletion in the APC gene. The genomic locus is chr5:112173265_112173266, with the specific variant designated as c.1974_1975del (p.Asn659Glnfs*14). Red arrows indicate the site of the deletion, where double peaks are observed in both forward and reverse sequencing traces, confirming the presence of a heterozygous deletion mutation.

In light of the aforementioned findings, we proceeded with molecular genetic testing on the patient. The results indicated a heterozygous deletion in the APC gene; the chromosomal location was chr5: 112173265_112173266. The inheritance pattern is autosomal dominant (AD). The condition is identified as FAP 1 [MIM:175100] and Gastric Adenocarcinoma and Proximal Polyposis of the Stomach [MIM:619182] (Figure 3).

Following confirmation of the diagnosis, the patient received five cycles of combination therapy consisting of bevacizumab (300 mg, day 1) for anti‐angiogenesis, oxaliplatin (150 mg, day 1), and capecitabine (1.5 g twice daily, days 1–14). In April 2024, reassessment revealed progression of pulmonary lesions. The patient was subsequently treated with six cycles of the modified FOLFIRI regimen combined with bevacizumab, consisting of bevacizumab (300 mg, day 1), irinotecan (240 mg, day 1), leucovorin (570 mg, day 1), bolus fluorouracil (570 mg, day 1), and continuous infusion fluorouracil (3.25 g over 48 h). In July 2024, CT imaging revealed progressive disease with hepatic, pulmonary, and pancreatic metastases. Consequently, on July 29, 2024, the patient was initiated on combination therapy consisting of sintilimab (200 mg, day 1), regorafenib (80 mg once daily), and trifluridine/tipiracil (45 mg twice daily) for two treatment cycles. In September 2024, the patient was readmitted for disease evaluation, which revealed disease progression. During hospitalization, she developed fever, scleral icterus, and skin jaundice. Cholangitis was suspected, and the patient's symptoms improved following initiation of anti‐infective therapy. On October 17, 2024, the patient received two cycles of chemotherapy with raltitrexed (4 mg). She subsequently underwent radioactive seed implantation for liver metastases on October 18, 2024, and for pancreatic metastases on November 8, 2024. On December 17, 2024, disease progression was confirmed upon re‐evaluation, and the patient was started on anlotinib as targeted therapy. However, due to intolerable drug‐related abdominal pain, the patient discontinued anlotinib after 3 days and subsequently refused further anti‐tumor treatment. Supportive and symptomatic care was provided thereafter. Disease progression was confirmed again on March 17, 2025, and the patient died in May 2025 (Table 1).

**TABLE 1:** Timeline of the disease and treatment.

Timeline	Diagnosis	Treatment	Outcome
2014	Papillary thyroid carcinoma (no additional clinical data available)	Previous thyroid surgery for papillary thyroid carcinoma, with undocumented extent of resection and nodal dissection	Postsurgical hypothyroidism following thyroid carcinoma treatment; No evidence of disease recurrence
January 2023	Multiple gastric polyps (missing pathology)	NA	NA
November 22, 2023	Malignant colonic obstruction at splenic flexure, suspected colon cancer (awaiting histopathology)	Self‐expandable metal stent (SEMS) placement for malignant colonic obstruction	Malignant splenic flexure obstruction with successful treatment response
December 13, 2023	Obstructive jaundice, etiology undetermined	PTBD	Jaundice resolution (within 4 weeks post‐treatment)
December 16, 2023	Colon adenocarcinoma, pT3N2M1c, Stage IV (AJCC 8th Edition), with synchronous liver and lung metastases, proficient mismatch repair (pMMR) by immunohistochemistry (MLH1+, PMS2+, MSH2+, MSH6+), EpCAM‐positive, Ki‐67 proliferation index 90% (hotspot)	Completed 5 cycles of combination therapy (XELOX‐A regimen: bevacizumab 300 mg IV infusion day 1, oxaliplatin 150 mg IV infusion day 1, capecitabine 1.5 g PO twice daily days 1–14, every 3 weeks)	PFS:3.8 months; Radiologically confirmed disease progression (increase in pulmonary lesions)
April 9,2024	Colon adenocarcinoma, pT3N2M1c, Stage IV (AJCC 8th Edition), with synchronous liver and lung metastases, proficient mismatch repair (pMMR) by immunohistochemistry (MLH1+, PMS2+, MSH2+, MSH6+), EpCAM‐positive, Ki‐67 proliferation index 90% (hotspot)	Completed 6 cycles of combination therapy(FOLFIRI‐A regimen: bevacizumab 300 mg IV infusion day 1, irinotecan 240 mg IV infusion day 1, leucovorin 570 mg IV infusion day 1, fluorouracil 570 mg IV infusion day 1 followed by 3250 mg continuous IV infusion over 46–48 h, every 2 weeks)	PFS:3.6 months; Radiologically confirmed disease progression (newly developed pancreatic metastasis)
July 29,2024	Colon adenocarcinoma, pT3N2M1c, Stage IV (AJCC 8th Edition), with synchronous liver, lung, and pancreatic metastases, proficient mismatch repair (pMMR) by immunohistochemistry (MLH1+, PMS2+, MSH2+, MSH6+), EpCAM‐positive, Ki‐67 proliferation index 90% (hotspot)	Completed 2 cycles of combination therapy (Sintilimab 200 mg IV infusion day 1 + regorafenib 80 mg PO once daily + trifluridine/tipiracil 45 mg PO twice daily days 1–5, every 4 weeks)	PFS:2.9 months; Radiologically confirmed disease progression (enlargement of liver metastases in colorectal cancer)
October 17,2024	Colon adenocarcinoma, pT3N2M1c, Stage IV (AJCC 8th Edition), with synchronous liver, lung, and pancreatic metastases, proficient mismatch repair (pMMR) by immunohistochemistry (MLH1+, PMS2+, MSH2+, MSH6+), EpCAM‐positive, Ki‐67 proliferation index 90% (hotspot)	CT‐guided 125I brachytherapy for liver metastases + CT‐guided 125I brachytherapy for pancreatic metastasis + Completed 2 cycles of raltitrexed‐based chemotherapy (4 mg IV infusion day 1, every 3 weeks)	PFS:1.8 months; Radiologically confirmed disease progression with both enlargement of existing liver metastases and new hepatic lesions in colorectal cancer
December 17,2024	Colon adenocarcinoma, pT3N2M1c, Stage IV (AJCC 8th Edition), with synchronous liver, lung, and pancreatic metastases, proficient mismatch repair (pMMR) by immunohistochemistry (MLH1+, PMS2+, MSH2+, MSH6+), EpCAM‐positive, Ki‐67 proliferation index 90% (hotspot)	Anlotinib hydrochloride 10 mg PO qd (21‐day cycle with 14‐day on/7‐day off)	PFS:0.1 months; Self‐discontinued medication secondary to grade 3 treatment‐emergent abdominal pain(CTCAE v5.0)
December 20,2024	Colon adenocarcinoma, pT3N2M1c, Stage IV (AJCC 8th Edition), with synchronous liver, lung, and pancreatic metastases, proficient mismatch repair (pMMR) by immunohistochemistry (MLH1+, PMS2+, MSH2+, MSH6+), EpCAM‐positive, Ki‐67 proliferation index 90% (hotspot)	Symptom‐directed supportive management	PFS:2.9 months; Radiologically confirmed disease progression with both enlargement of existing liver metastases and new hepatic lesions in colorectal cancer
March 17,2025	Colon adenocarcinoma, pT3N2M1c, Stage IV (AJCC 8th Edition), with synchronous liver, lung, and pancreatic metastases, proficient mismatch repair (pMMR) by immunohistochemistry (MLH1+, PMS2+, MSH2+, MSH6+), EpCAM‐positive, Ki‐67 proliferation index 90% (hotspot)	Symptom‐directed supportive management	PFS:1.8 months; Final follow‐up: May 2025 (patient deceased)

## 3 Discussion

In our case report, the patient's prior history of thyroid cancer (TC) warrants particular attention. APC gene mutations can lead to FAP; however, not all FAP patients develop thyroid cancer (TC). A meta‐analysis encompassing up to 9821 FAP patients reported the prevalence of coexisting TC, benign thyroid nodules, and thyroid endocrine disorders to be 2.6% [95% CI 1.3–4.8], 48.8% [95% CI 33.8–64.0], and 6.9% [95% CI 4.5–10.3], respectively [9]. In comparison, the incidence of FAP in live births is estimated at approximately 1 in 30,000 to 1 in 7000 [10]. These data clearly indicate that the co‐occurrence of FAP and TC is relatively rare. Furthermore, the data from the aforementioned meta‐analysis indicate a substantial prevalence of thyroid disorders—encompassing malignancies, benign nodules, and endocrine diseases—among patients with FAP. Previously, the American Gastroenterological Association recommended annual thyroid ultrasound screening for patients with FAP. However, Chenbhanich et al. have suggested that thyroid ultrasound screening should be targeted specifically toward FAP patients who are at high risk for thyroid cancer (TC), rather than universally applied to all FAP patients. This recommendation is based on several factors: (a) FAP‐associated thyroid cancer (TC) typically presents as a less aggressive form of papillary thyroid carcinoma, with an extremely low mortality rate. Patients often succumb to other causes rather than TC. (b) Routine thyroid ultrasound screening may not only impose a burden and cause significant anxiety for patients undergoing surveillance, but also, due to its relatively low specificity, lead to a high rate of false‐positive results. (c) There is currently a lack of cost‐effectiveness analyses evaluating the use of ultrasound surveillance specifically for FAP‐associated thyroid cancer. It is important to note that high‐risk FAP patients for thyroid cancer (TC) are specifically defined as young females under the age of 31 with APC gene mutations located at the 5ˊ end of the gene. This risk stratification is based on the following evidence: (a) Approximately 95% ± 3.9% of patients with FAP‐associated TC are female; (b) The mean age at FAP diagnosis is 29 ± 11.4 years, and the average age of TC onset is 31 ± 11.8 years; (c) Studies have shown that mutations in the 5ˊ end of the APC gene, particularly near codon 938, account for approximately 44.2% of FAP‐TC cases, whereas only 17.5% of mutations in the general FAP population occur in this region. This significant enrichment highlights a strong genotype–phenotype correlation, underscoring the increased risk of TC associated with 5ˊ end APC mutations in FAP [9]. Although the association between APC gene mutation location and the FAP‐TC phenotype has been established, the underlying molecular mechanisms remain unclear. The rarity of FAP‐TC results in limited sample sizes—only 52 cases have been reported [9]—which may contribute to potential bias in the observed associations and limits the statistical power for definitive conclusions. Additionally, in patients with FAP‐associated thyroid cancer (FAP‐TC), there is a significant disparity in the male‐to‐female ratio, suggesting the potential involvement of epigenetic or environmental factors—such as hormonal influences—in the pathogenesis of FAP‐TC. Comprehensive studies examining the roles of epigenetics, environmental exposures, and hormonal influences could provide valuable insights into the mechanisms driving FAP‐TC development.

The aforementioned discussion is based on cases where TC is identified after the diagnosis of FAP. In fact, approximately one‐third of patients develop TC prior to the diagnosis of FAP [11]. Our patient is a representative example of this clinical scenario. This highlights the importance of considering FAP in the differential diagnosis of individuals presenting with early‐onset or multiple adenomas, particularly when there is a personal or family history of extracolonic malignancies such as thyroid cancer. The patient has a family history of colorectal cancer and was diagnosed with papillary thyroid carcinoma (PTC) at the age of 18, for which she underwent surgical treatment. Postoperative follow‐up showed no evidence of TC recurrence, indicating that the likelihood of death due to TC is low in this patient. Notably, the histological type of the patient's TC is papillary carcinoma. Studies have shown that approximately 83.3% of FAP‐associated thyroid carcinomas (TC) exhibit the histological features of papillary thyroid carcinoma (PTC) [9]. Among these, the proportion of the cribriform‐morular variant of papillary thyroid carcinoma (CMV‐PTC) ranges from 0% to 12.5% [9]. However, some authors estimate its prevalence in FAP‐TC patients to be as high as 80%, suggesting that this pathological variant may serve as a surrogate marker for the diagnosis of FAP [9]. This variant is exceedingly rare, accounting for only 0.1%–0.2% of all PTC cases. In patients with FAP and CMV‐PTC, about half present initially with thyroid cancer, and more than half develop bilateral multicentric tumors. Furthermore, most CMV‐PTCs appear benign on ultrasound examination [11]. Unfortunately, we were unable to obtain histopathological images of the patient's thyroid cancer tissue, which precludes definitive confirmation of whether it represents the CMV‐PTC.

The patient was diagnosed with FAP at the age of 30, which is consistent with previous studies showing that FAP patients are typically diagnosed before 31 years of age. However, subsequent genetic testing revealed that the patient harbors a deletion mutation in exon 16 of the APC gene at position c.1974_1975del, resulting in an amino acid substitution from Asn to Gln at position 659, followed by a frameshift leading to a premature stop codon after the 14th amino acid downstream. This finding contrasts with the mutations near codon 938 at the 5′ end of the APC gene as highlighted by Chenbhanich et al. [9]. We speculate that this discrepancy could be attributed to selection bias. The patient has ultimately succumbed to progressive colorectal cancer. Had she undergone genetic testing at the age of 18 upon diagnosis of papillary thyroid carcinoma (PTC), leading to an earlier diagnosis of FAP and subsequent timely interventions, her outcome might have been different. This case highlights the importance of considering APC gene mutation testing in young patients diagnosed with PTC, particularly when there is a personal or family history suggestive of FAP. Early identification of FAP through molecular screening could enable proactive surveillance and preventive measures—such as prophylactic colectomy or intensive colonoscopic monitoring—potentially averting the development of advanced colorectal cancer.

FAP is an incurable cancer predisposition syndrome that requires intensive endoscopic surveillance and prophylactic surgical intervention for the colon, rectum, and duodenum to prevent malignant transformation. The optimal management strategy involves definitive resection of the primary disease site. Surgical options include total proctocolectomy with permanent ileostomy, total colectomy with ileorectal anastomosis, or total colectomy with rectal mucosectomy and ileal pouch‐anal anastomosis (IPAA) [2]. Unfortunately, our patient was diagnosed with FAP at an advanced stage, already presenting with hepatic, pulmonary, and lymph node metastases, rendering her ineligible for curative surgical resection. Moreover, the patient refused to undergo genetic testing of the tumor tissue.

For this patient's treatment plan, we adhered to the CSCO guidelines, which emphasize the necessity of genetic testing (RAS, BRAF) in metastatic colorectal cancer. However, this recommendation conflicted with the patient's refusal to undergo testing. In the absence of information regarding RAS or BRAF mutation status, our clinical strategy was shifted from a precision medicine approach to a “default safest and most universally applicable” mode—effectively adopting a “worst‐case scenario” principle. We thus assumed potential RAS or BRAF mutations and excluded anti‐EGFR agents upfront, opting instead for a chemotherapy plus anti‐VEGF regimen. When this initial strategy proved ineffective, we attempted a combination of sintilimab, regorafenib, and trifluridine/tipiracil. However, the disease continued to progress with declining patient tolerance. We subsequently switched to raltitrexed chemotherapy, yet disease progression persisted. Ultimately, we administered anlotinib as a final attempt to achieve disease control. In addition, our treatment strategy included endoscopic stenting to relieve intestinal obstruction and radioactive seed implantation for metastatic lesions. However, the therapeutic response was suboptimal, and the patient ultimately succumbed to progressive disease, with an overall survival of approximately 19 months from the time of FAP diagnosis (Table 1).

A notable limitation in this case is the patient's decision to decline comprehensive genetic profiling, which includes testing for RAS (KRAS and NRAS) and BRAF mutations. This omission considerably constrains the interpretation of treatment outcomes and reflects a common challenge in clinical scenarios involving advanced cancer. The absence of biomarker data precludes a biologically guided selection of targeted therapies. For instance, anti‐EGFR agents such as cetuximab or panitumumab are exclusively indicated for patients with RAS wild‐type tumors. Without confirmed RAS status, the potential benefits of these therapies remain unknown, and their empirical use could have resulted in unnecessary toxicity without clinical benefit. From a broader perspective, this case underscores socioeconomic and psychological barriers that may prevent patients from accessing precision oncology. It highlights an ongoing need to improve patient education, mitigate financial burdens, and integrate shared decision‐making to optimize adherence to evidence‐based care pathways. In summary, the lack of molecular characterization limits the generalizability and mechanistic insight of this case report, while emphasizing the critical importance of biomarker testing in modern oncology practice.

We attribute the suboptimal treatment response to several factors: (a) Patient‐specific reasons: The patient did not undergo genetic testing of the tumor tissue, making it difficult to formulate targeted treatment. Moreover, the patient discovered the tumor at a late stage, with multiple metastases throughout the body and a heavy tumor burden. (b) Unique molecular pathogenesis—APC gene mutation/Wnt pathway constitutive activation: The root cause of FAP is mutations in the APC gene. The APC protein serves as a critical negative regulator of the Wnt/β‐catenin signaling pathway. Loss of APC function leads to abnormal accumulation of β‐catenin and subsequent constitutive activation of the Wnt pathway. This persistent activation drives cellular proliferation and inhibits differentiation, acting as both the “initiating factor” and “sustained driving force” for the formation and malignant transformation of FAP polyps [12]. Traditional chemotherapy agents may be insufficient in effectively targeting or overcoming the core survival and proliferative advantages conferred by this oncogenic signaling pathway (Wnt). Therefore, therapeutic strategies specifically targeting aberrant Wnt signaling may be more effective, which warrants further validation. (c) Impact of chromosomal instability (CIN): The carcinogenesis process in FAP‐CRC follows the classical adenoma‐carcinoma sequence model, where APC mutations initiate constitutive activation of the Wnt/β‐catenin signaling pathway and chromosomal instability (CIN). This subsequently drives mutations in other CIN genes (APC, RAS, SMAD4, and TP53) and DNA damage repair genes (SUZ12, KMT2D, BCLAF1, RUNX1, and ARID1B), ultimately leading to microsatellite‐stable (MSS) tumor development. The vast majority of FAP‐CRCs exhibit MSS phenotype [13]. (d) Tumor Microenvironment and Therapeutic Resistance: APC mutation‐induced chromosomal instability (CIN) triggers chronic cGAS‐STING activation, reprogramming downstream signaling to establish an immunosuppressive tumor microenvironment (TME) that promotes metastasis [14] and confers therapeutic resistance. (e) Limitations of Clinical Studies: FAP‐associated cancers are relatively rare, making it difficult to conduct large‐scale randomized controlled trials (RCTs) to establish optimal chemotherapy regimens. As a result, current treatment strategies are largely based on retrospective studies and clinical experience. These data consistently indicate that FAP‐related tumors often exhibit lower‐than‐expected response rates to standard chemotherapy, with limited survival benefit and, in some cases, rapid disease progression.

Nevertheless, upon the development of FAP‐associated colorectal cancer (FAP‐CRC), surgical resection remains the treatment of choice whenever feasible. However, it is important to emphasize that surgery does not constitute a cure for FAP, as the underlying genetic predisposition persists. Even after prophylactic or therapeutic colectomy, patients remain at significant risk for developing extracolonic malignancies, including duodenal cancer, gastric cancer, and thyroid cancer—beyond the well‐recognized risk of desmoid tumors. To date, various agents including celecoxib, sulindac, aspirin, fish oil, ascorbic acid (or vitamin C), and combinations such as sulindac/difluoromethylornithine (DFMO), celecoxib/DFMO, and sulindac/erlotinib have been investigated for their potential benefits in slowing disease progression and inducing polyp regression in FAP. Although these therapeutic options have shown some promise in both preclinical models and clinical trials, their overall efficacy has not been entirely satisfactory [15]. Several studies have explored the repurposing of existing drugs to identify agents capable of delaying polyp progression, thereby reducing the risk of colorectal cancer development or promoting tumor cell differentiation in FAP. Aberrant proliferation due to APC loss is a hallmark of the early stages of colorectal carcinogenesis. Notably, APC‐driven tumorigenesis has been shown to depend on the activation of mTORC1 (mechanistic target of rapamycin complex 1). Rapamycin, an inhibitor of mTOR (mechanistic target of rapamycin), effectively suppresses mTORC1 signaling. By inhibiting this critical downstream pathway, rapamycin can counteract the hyperproliferative state induced by APC deficiency [15]. Preclinical animal studies have already demonstrated this effect [16], and there have been reports of successful treatment with rapamycin in two FAP patients [17]. These findings indicate that rapamycin, as a highly promising agent, may offer significant therapeutic benefits for individuals at high risk of colorectal cancer.

## 4 Conclusion

We identified a deletion mutation, c.1974_1975del, in exon 16 of the APC gene. Mutations in the APC gene lead to FAP and can also result in extracolonic malignancies, including thyroid cancer. In clinical practice, when encountering young patients with thyroid cancer, it is crucial to thoroughly investigate family history for hereditary conditions. Gastrointestinal endoscopy and ophthalmological screening should be conducted, along with further molecular genetic testing if necessary. Additionally, when patients present with multiple gastric or colonic polyps, mutations in either the APC or MUTYH genes should be considered. Specifically, for young female patients under 31 years of age diagnosed with FAP due to mutations at the 5′ end of the APC gene, annual thyroid ultrasound screening is recommended. This comprehensive approach will aid in early detection and management of associated malignancies, thereby improving patient outcomes.

## Author Contributions

Conceptualization: Ren Yijing, Wang Wenjun, Zhou Ping, Liao Wei. Investigation and data curation: Gao Xiang, Xing Kongling, Chen Yunxia. Validation and visualization: Ren Yijing, Wang Wenjun, Zhou Ping, Liao Wei. Writing – original draft: Ren Yijing, Wang Wenjun. Writing – review and editing: Zhou Ping, Liao Wei. Project administration: Zhou Ping, Liao Wei.

## Consent

We had previously obtained the patient's written consent for the publication of any potentially identifiable images or data included in this article.

## Conflicts of Interest

The authors declare no conflicts of interest.

## Data Availability

The data that support the findings of this study are available on request from the corresponding author. The data are not publicly available due to privacy or ethical restrictions.
